# Active and Assisted Living Ecosystem for the Elderly

**DOI:** 10.3390/s18041246

**Published:** 2018-04-17

**Authors:** Isabel Marcelino, Rosalía Laza, Patrício Domingues, Silvana Gómez-Meire, Florentino Fdez-Riverola, António Pereira

**Affiliations:** 1 INOV INESC INOVAÇÃO Instituto de Novas Tecnologias—Delegação de Leiria, 2411-901 Leiria, Portugal; isabel.marcelino@ipleiria.pt (I.M.); antonio.pereira@ipleiria.pt (A.P.); 2 School of Technology and Management, Computer Science and Communications Research Centre, Polytechnic Institute of Leiria, 2411-901 Leiria, Portugal; patricio.domingues@ipleiria.pt; 3 ESEI: Escuela Superior de Ingeniería Informática, University of Vigo, 32004 Ourense, Spain; rlaza@uvigo.es (R.L.); sgmeire@uvigo.es (S.G.-M.); 4 CINBIO: Centro de Investigaciones Biomédicas, University of Vigo, 36310 Vigo, Spain; 5 Grupo de Investigación SING, Instituto de Investigación Sanitaria Galicia Sur (IIS Galicia Sur), SERGAS-UVIGO, 36312 Vigo, Spain

**Keywords:** telemedicine, gerontechnology, home care monitoring systems, active assisted living, remote health monitoring

## Abstract

A novel ecosystem to promote the physical, emotional and psychic health and well-being of the elderly is presented. Our proposal was designed to add several services developed to meet the needs of the senior population, namely services to improve social inclusion and increase contribution to society. Moreover, the solution monitors the vital signs of elderly individuals, as well as environmental parameters and behavior patterns, in order to seek eminent danger situations and predict potential hazardous issues, acting in accordance with the various alert levels specified for each individual. The platform was tested by seniors in a real scenario. The experimental results demonstrated that the proposed ecosystem was well accepted and is easy to use by seniors.

## 1. Introduction

Society is changing every day, leading to new realities and new challenges. According to the United Nations report “World Population Ageing 2013”, human life expectancy is rising [1]. The emancipation of women and worldwide emigrations are other examples in society that have led to a reorganization of relationships among family members [2]. The fact that people are less available to give proper support to elderly family members, together with the high costs of hospitalization and nursing homes, has led to a new challenge: how to improve the quality of life for the elderly while also reducing costs.

In such a situation, the study presented in [3] analyses different successful aging concepts such as physical health, independence, reception of emotional care and economic security. The solution involves providing a secure environment in the homes of elderly people, assuring independence, monitoring health and, above all, being able to administer appropriate emotional care. To address these challenges, several emerging technologies and concepts play an empowering role [4]. Gerontechnology is one of these pillar concepts [5,6,7], which, additionally, aims to strengthen communication methods within the senior population [8].

The concept of Active Assisted Living (AAL) in [9] is understood as a means of prolonging the time people can live in their preferred environment (usually at home) and avoid social isolation by giving them proper support. Wireless technology is a set of devices connected by optical or infrared radio frequency that enable ubiquitous networking for anyone, any time and anywhere. Wireless Sensor Networks (WSN) inherit wireless abilities, adding a sensing skill. They can be used for monitoring environments, leveraging smart homes [10,11]. Another technology connected to gerontechnology is Body Area Networks (BAN), which provide low cost healthcare solutions through continuous monitoring, leading to the early detection of abnormal conditions [12,13]. BAN can be defined by a set of low power acquisition devices (sensor nodes) that gather vital parameters from an organism. These sensors may collect and also respond to certain circumstances [14,15]. It is also important to obtain the physical location of the elderly individual in order to provide an effective response in cases outside the norm, sending specialized help to an accurate location, without neglecting security and ethical issues raised by these kinds of features [16]. This tracking may be achieved with Radio Frequency Identification (RFID) tags and Global Positioning System (GPS) personal locators.

It is our belief that the above discussed challenges can be addressed through the use of several technologies. However, it is a well-known fact that the senior population frequently suffers from technological illiteracy. Therefore, another challenge that must still be addressed today is: how to promote the usage of new technologies by the senior population, in order to improve their quality of life? To this end, we have developed an innovative solution focused on the needs of the senior population, addressing issues such as (i) having a simple and non-invasive way to monitor seniors, either by monitoring vital parameters or monitoring the environment and their location; (ii) promoting a user-centered design with a primary focus on inclusive design, gerontodesign and standards of usability and accessibility; (iii) providing a large range of services to reassure emotional comfort, improve their health condition, increase communications and reduce physical barriers, as well as provide new forms of joyful entertainment in their lives, ensuring an active aging process.

Our research approach was organized as follows. First, an analysis of the state of the art was carried out, along with several semi-structured interviews used as requirement surveys. These interviews allowed us to identify some gaps in existing solutions and to propose a support service platform, eServices, for non-invasive continuous surveillance and social inclusions. In [17], we presented a brief overview of eServices, but our research work is shown in detail in the current paper, where eServices features are exhaustively explained along with the modules included in the eServices solution. Additionally, we have enlarged our study regarding related work, while seeking new aspects that may enrich our contribution. We have also conducted more acceptance tests in order to infer significant conclusions.

While this section has introduced and laid out the motivations for the work, the remainder of the paper is organized as follows: related studies and unsolved issues are stated in Section 2. Section 3 describes eServices as a solution to suppress the identified limitations, followed by experiments and results explained in Section 4. Finally, conclusions and future work are presented in Section 5.

## 2. Related Works

As mentioned in the previous section, world life expectancy is increasing. Senior people need special care due to their age-related impairments. In addition to physical issues, extreme loneliness and isolation also need to be addressed. Because of the inverse trend between active and retired people, governments will have massive pension expenses. Medical care expenses are also going to increase due to the impaired health status of most elderly people. Therefore, a major challenge will be to provide proper support and well-being to the senior population with reduced financial resources. There are several projects in gerontechnology and other overlapping areas. While some are academic and others commercial, all are intend to contribute to this emerging problem. There are numerous smartphone apps dedicated to health issues, either to record or monitor vital signs or to promote physical exercise and posture. Additionally, specialized phone attachments or wireless sensors are able to extend functionalities that are not part of a phone’s standard functions [18].

Microsoft has a solution to measure gait and balance using Kinect for Xbox 360 [19], designed for older people. This shows concern for the senior population and represents an attempt to contribute to improving the quality of life of the elderly. There are also several companies developing commercial sensors to gather vital parameter information. Some of them use external sensors, others textile sensors or even implanted sensors. An excellent overview of the current status and future perspectives of wearable systems related to healthcare is presented in [20]. Commonly, these sensors transmit information to a central point where further processing and analysis is done [21].

From a complementary perspective, other companies are developing alarm services, such as panic buttons on bracelets, necklaces or mobile phones, associating them with telecare service. Some current examples available in Portugal are “Cruz Vermelha Portuguesa Teleassistência” [22] and “Portugal Telecom Teleassistência” [23]. These services have twenty-four hour call centers with specialized support and also provide SOS buttons on necklaces as a paid service. With regard to the costs associated with these solutions, “Cruz Vermelha Portuguesa Teleassistência” presented a price list in 2018 ranging from €5 to €41 per month, according to the subscription options. On the other hand, the only associated costs for “Portugal Telecom Teleassistência,” a service provided by a foundation, are those related to having a landline or mobile phone and any corresponding call charges.

PANGEA (Platform for Automatic coNstruction of orGanizations of intElligent Agents) is an example of an agent platform to develop open multi-agent systems, such as supervision architectures for medical care, specifically focused on dependent people. Due to its service orientation, different tools modeled with agents that consume web services can be integrated and operated from the platform, regardless of their physical location or implementation [24,25]. A solution using PANGEA to monitor, detect and prevent anomalous situations is presented in [26]. It aims to provide an affordable solution, including hardware and software, to perform ECG measurements, monitoring through accelerometers and Wi-Fi networks and establishing an interaction with the system through the use of a television.

A multi-agent information fusion system to manage data from a WSN in a residential home is presented in [27]. Its purpose is to improve the healthcare of dependent people in geriatric residences and in their own homes, focusing on information fusion techniques.

An overview of technology-supported personal care research activities is made in [28], which presents an exhaustive list of projects. One of these projects is PERSONA (Perceptive Spaces promoting Independent Aging). Its goal is to develop a scalable open standard technological platform to build a broad range of AAL services, allowing ad hoc addition of services.

Platforms and reference architectures for AAL systems are also approached in [29]. OASIS (Open architecture for Accessible Services Integration and Standardization) is one of the mentioned platforms that also follows a service-oriented approach [30]. The key motivation is for ontologies to ensure machine-level interoperability and for glossing and document concepts to be understood and equally interpreted by service developers and end users [31]. Another example worth mentioning is AAL4ALL, a Portuguese project that comprises a 35-entity consortium, each with the common goal of developing a product and service ecosystem for the industrial generalization of AAL [32]. The UserAccess project was created as an AAL4ALL product and a test case for the viability of the entire AAL4ALL project [33]. The specifications for UserAccess include the use of the Tomcat apache server with the REST communications protocol implemented in a multi-agent system, ZigBee sensors for environment monitoring and the WEKA package for generating the final data classification and association.

The study in [34] refers to six European projects towards a future where people can have an active role managing their own healthcare, especially seniors: Complete Ambient Assisted Living Experiment (CAALYX), Enhanced Complete Ambient Assisted Living Experiment (eCAALYX) [35], COGKNOW, EasyLine+, I2HOME and SHARE-it. The intention is to allow the elderly to remain as independent as possible, reducing nursing admission costs and increasing virtual healthcare providers and career visits. Additionally, providing information to end users will allow them to be more aware of their conditions and much more assertive in their lifestyle.

UniversAAL has developed an open platform mostly focused on assuring interoperability and standardization that allows plug-and-play for AAL applications and devices [36]. This platform is the result of observing that many current middleware architectures (connecting services and service providers) have missing standards in some AAL fields of application such as remote maintenance, terminology and ontology, emergency calls and procedures. They also identify various domains of AAL such as eHealth, home entertainment, home automation, household appliances, energy control and saving. A comprehensive resource containing all the information and tools to begin developing AAL applications using the UniversAAL platform is provided in [37]. This project has evolved into UniversALL IoT, which seeks to: (i) plug multiple sensor and actuator technologies, including ZigBee, ZWave, EnOcean, KNX and Open Home Automation Bus (OpenHAB); (ii) enable external communications with other solutions by using ontologies; and (iii) provide an open platform compatible with numerous deployment architectures, either using Java version on Open Services Gateway Initiative (OSGi), and embed on everything from a PC to Raspberry Pi, or using it as an app on Android, or accessing it as a RESTful API [38].

Continuous and recent calls in the Active and Assisted Living Programme-ICT for aging well indicate that there is still a long road ahead in order to achieve viable and effective solutions; namely, call 2015, which was focused on living actively and independently at home, call 2016, which was focused on living well with dementia or more recently, call 2017 for AAL packages/integrated solutions. As a result of these calls, many research issues and projects were successfully addressed [39], such as: (i) Active@Home, which appears as an interactive video game-based training with dance and Tai Chi elements; (ii) Brain@Home, which aims to prevent and detect cognitive decline early through serious gaming; (iii) Time and Skill Bank for Active Aging (TSBank), which consists of a time bank platform developed for the senior population to trade their time and skills; or (iv) Medguide to support seniors with dementia with their medication adherence through the use of smart pill boxes and social networking.

Despite the existence of several successful initiatives in the AAL domain, several key aspects are still unresolved such as: (i) not taking into account the overall needs of the elderly person; (ii) excessively complex and invasive approaches; (iii) not assuming that end users can be partially/completely technologically illiterate; (iv) the absence of a user-centered design approach; (v) being unreliable and reactive; (vi) an application of the same response/output for every individual; (vii) occasional disregard for security and ethical issues; (viii) lacking standards and protocols raising integration issues; (ix) being non-extensible; (x) lacking implement solutions with any service, accessible from any location, on any device, at any time, to every policy; and (xi) a high cost. We have broken down all these issues by identifying fundamental characteristics and comparing their presence in the above-mentioned platforms. Table 1 collects the treatment of these key features in previously-developed solutions (i.e., PERSONA, OASIS, UniversAAL, AAL4ALL and eCAALYX), which are closely related to the goals of eServices.

To overcome some of the above identified issues, the next section introduces and explains in detail our eServices platform.

## 3. eServices: Elderly Support Service Platform

As mentioned in the previous section, eServices was developed to respond to all key features that this research team has pointed out to be vital in solutions to improve the quality of life and well-being of the elderly. The main architecture of eServices combines end user clients, service providers and the middleware platform between end users and service providers.

The end user client module has three components: biosensors to monitor basic life sign sensing; environment sensors; and a service access device to interact with the provided services. An important guideline for the development of these components is to ensure they are as non-invasive as possible and also extremely simple. The middleware platform contains service catalogue, system administration and event management modules. In order to grant scalability, high availability and reliability, all the components in the middleware platform have replication and balanced distribution methods. Services providers will register their service in the service catalogue present in the middleware platform. The following subsections explain in detail the three major components of eServices: end user client, middleware platform and services provider.

### 3.1. End User Client

**Figure 1:**
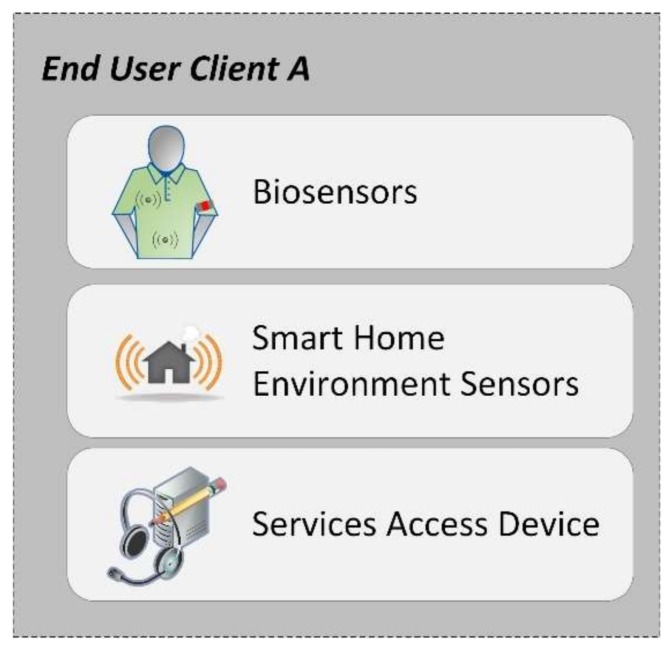
End user client.

The end user client is an input module for the middleware platform. It is structured as three sub-components. As seen in Figure 1, the three sub-components are biosensors, environment sensors and the service access device.

The main function of the end user client is to gather vital parameters through BAN, to record events such as fire, gas, movement detection, burglar and intruder alarms, etc., and to allow interaction with the device where some services are going to be available. For that purpose, whether in the private homes of seniors, nursing homes or outdoors, each individual will have a biosensor kit together with a virtual service access device.

A minimal approach for the biosensor kit is to only include a modular sensorial node fitted with a fall detection sensor [40]. Although this approach is successful for the detection of falls, it might not recognize other important events. A more robust approach requires three of these sensors, allowing the full characterization of the movements of the monitored elderly person. Detection of movement anomalies is done by periodically comparing the current set of movements with the expected regular pattern. Whenever a deviation from the regular pattern is detected, e.g., the elderly patient fails to move for a long period of time and/or has failed to leave the house during the entire morning as he/she usually does, the system triggers appropriate measures. Other parameters can be monitored if the need arises. For example, a heartbeat monitor can be fitted on the custom-made sensorial node for individuals with a heart condition requiring constant monitoring. Other sensors can be fitted on a sensorial node.

Regarding data exchange, one of the sensorial nodes fitted on a monitored individual functions as the communication coordinator. This sensorial node gathers the data of all the other body-fitted sensors and sends them either to a gateway node or to a smartphone that acts as a gateway. The sensorial nodes exchange data with the coordinator through the IEEE 802.15.4 Low Rate Wireless Personal Area Networks (LRWPAN), a standard that is the base for the ZigBee specification. Namely, the ZigBee specification extends the IEEE 802.15.4 standard by adding network and security layers and an application framework [41]. The coordinator node communicates with the gateway node through the IP protocol. A detailed description of the used BAN is given in [40], although it should be noted that a newer version of the sensorial nodes is currently being developed using the Bluetooth Low Energy communication protocol to further reduce power consumption.

The main intention in this approach is to allow users to have a small and non-invasive way of being monitored, while allowing for the addition of new sensors as needed. For example, in interviews, medical staff pointed out that some parameters are vital to monitor, especially in elderly people [42]. These essential parameters need to be in the base node. Other parameters requiring specific sensors will be coupled and chosen according to the needs identified for a given individual. This allows for a scalable solution, as well as “low cost” bases that can be updated afterwards by adding new sensors to the same node. The proposed solution is sensible and adapted to the limited resources provided by BAN and all the related security issues. One of the major concerns, in addition to monitoring basic vital signs such as heart rate or blood pressure, is to identify fall detection. Indeed, falls are pointed out as the most important issue to monitor regarding the senior population. As a major risk situation, falls must be detected as accurately as possible. This is challenging, since many fall detection techniques are prone to confuse normal and benign human movements like sitting and lying down with falls, and thus issue false positives. Work regarding the accurate detection of falls is being done by a complementary research team and is not the main objective of this paper. More details are given in [40,43,44].

The monitoring of the environment is done by environmental sensors. These sensors can be split into two main groups: (i) presence detection sensors and (ii) disaster sensors. The set of presence detection sensors includes door traversal detectors and domestic appliance monitors. A door traversal detector triggers a monitoring event whenever an individual crosses a monitored door, while a domestic appliance monitor is coupled to a domestic appliance to detect activity. Currently, only TV sets are monitored by way of the instrumented remote control (on/off). Disaster sensors, as the name implies, aim to detect situations before they escalate into catastrophes. Examples include, among others, gas sensors to detect natural gas leaks, monoxide carbon detectors, flooding sensors and smoke detectors.

All the environmental sensors exchange data through the eServices platform, each one communicating directly with the platform. The communication protocol used depends on the sensor, with possible protocols including WIFI, Bluetooth Low Energy and Power over Ethernet (PoE). Location-based services (RFID and GPS solutions) are used to obtain location. This allows the system to call the closest healthcare/emergency provider for specialized help. Other information may be inferred from the location data, namely behavior patterns, which can be further processed to establish routines and deviations. According to [45], location may be used as a quasi-identifier, relating a pseudonym to a real person. It is important to point out that all the gathered information is cyphered and classified and that there are measures to reduce location-based quasi-identifiers.

In addition to BAN, the environment sensors and the location-based services, it is also important to have a device that allows the user to communicate and interact with the services provided in eServices. This subject is posteriorly addressed in the presented paper, but it is now important to mention the deep concern regarding the lack of easy interaction methods between seniors and technology. Our purpose is to give seniors the simplest possible interface. Advanced concepts such as inclusive design and gerontodesign are being explored to achieve the major goal of providing exactly what the target users expect and feel comfortable with. It is important to recognize aging as a physical limitation and to test the solutions and developed applications with real subjects, collecting expectations and concerns in order to produce a final solution that seniors can and will be able to use. For this purpose, Quality of Experience (QoE) measures and metrics play a vital role [46]. The ITU Telecommunication Standardization Sector (ITU-T) defines QoE as “the overall acceptability of an application or service, as perceived subjectively by the end-user” [47]. Collecting Mean Opinion Scores (MOS) in a sample target population, analyzing answers and performing some reengineering process will make it possible to overcome and significantly reduce some resistance between end users and application interaction. The interface must be as intuitive as possible to avoid the need for memorization. Moreover, the interaction needs to adapt to the (usually low) user experience, hiding errors and other unexpected behaviors of the system as much as possible. Indeed, it is expected that a large percentage of seniors will not have the ability to work around any IT problem, thus leading to feelings of insecurity or resistance towards the system.

Regarding the proceeding, we have implemented a web application using HTML5 (HyperText Markup Language), CSS3 (Cascading Style Sheets), JavaScript and PHP (Hypertext Preprocessor) technologies. Moreover, we have developed the application with a responsive design supporting any device (e.g., mobile, tablet and/or personal computer) and following W3C (World Wide Web Consortium) recommendations concerning usability and accessibility guidelines. We have also included a WebRTC (Web Real-Time Communication) API to allow real-time peer-to-peer audio, video and data directly between clients’ browsers, without the need for external components. For communication security, we have used the sipML5 API that uses SIP (Session Initiation Protocol) over WebSockets to communicate with the existing servers in the middleware platform.

### 3.2. Middleware Platform

As previously mentioned, the middleware platform will be the bridge between end users and service providers. This scalable solution aims to provide services on demand. Users will be able to request services that are not yet available. The middleware platform has three main features: services catalogue, system administration and event management.

Moreover, the middleware platform intends to include several services and follows standards that allow for communication between all services provided and consumed. All services developed for eServices, by either this research team or others, must meet the established communication standards. In this context, there are several healthcare standards that must be considered. Messaging formats common in healthcare are Health Level 7 (HL7) and Digital Imaging and Communications in Medicine (DICOM). For health information exchanges, the main standards are the Integrating Healthcare Enterprise (IHE) and Healthcare Information Technology Standards Panel (HITSP). However, it has been observed that several health monitoring solutions and projects in AAL do not respond to any protocol or standard [48]. There are small projects that have been conceived to address a specific narrow problem; however, they are marred with interoperability issues when end users try to cooperatively merge more than one of these projects to achieve wider needs. To guarantee the integration of any service, simple integration mechanisms are integrated in eServices, allowing for the smooth addition of new services.

Regarding the proceeding, we have configured two complementary servers: (i) a Linux server to host the IMS (IP multimedia subsystem) that manages users, services and access; and (ii) a speech recognition server over a Windows platform. The Linux server also hosts a LAMP (Linux, Apache, MySQL, PHP) server for the web application and MySQL databases. These databases are the openIMS database and a complementary “extended database” for storing information that is not initially included in the openIMS database (e.g., user detailed information and interface advanced options). The second server uses the Microsoft Speech Recognition Engine and .NET language to receive audio from the web application, translate it to the correct command and transmit it back to the web application.

#### 3.2.1. Services Catalogue

**Figure 2:**
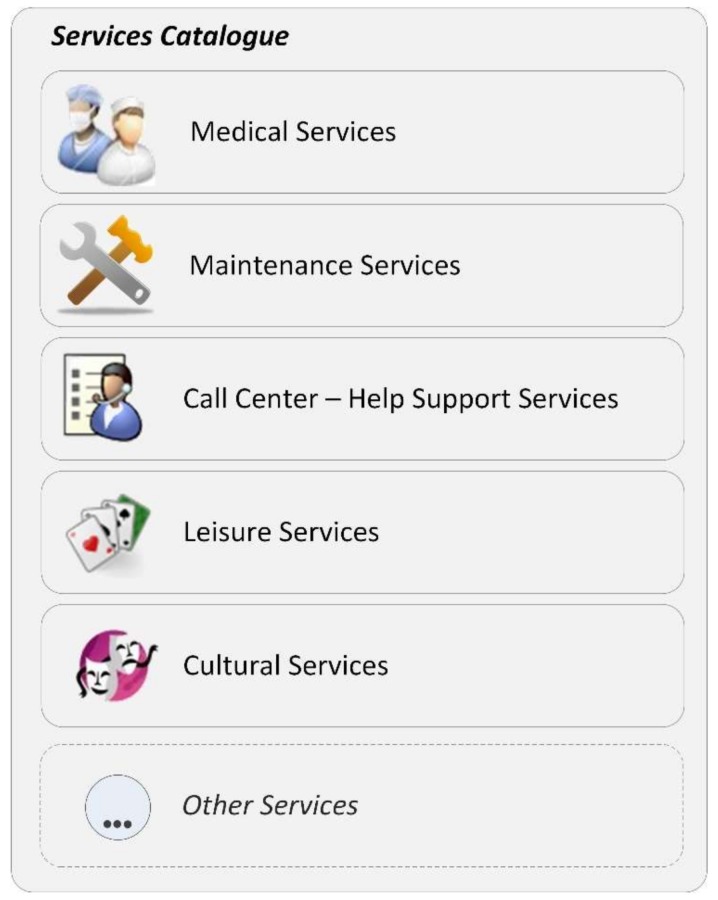
Service catalogue.

A services catalogue will be incorporated in the middleware platform providing a range of services in several categories. Figure 2 identifies some previously-identified categories: medical, maintenance, call center, leisure and cultural.

Under the medical category, services include virtual doctor appointments, which are performed through a web cam. The idea is not to replace face-to-face consultations, but to add this possibility for those who have less mobility, and also to reduce expenses in outings to health centers. The communication may be initiated from either end; for instance, if end users forget to take medication, caregivers can call to remind them to take it or give them other advice. The medical category also integrates the possibility to schedule appointments and exams. It also integrates reminders to take medication. Furthermore, the system allows for the addition of other relevant services under the medical category.

The maintenance category may be used to request repairmen (for plumbing, electricity), help in dealing with taxes (filling out forms, payments, etc.), request a taxi, ask for in-home hairdresser services, shopping or other small errands and request home delivery of medication. These small errands can also be achieved by the neighbor-to-neighbor time bank approach. This approach allows people to perform volunteer work and trade services. For instance, a former accountant can fill out the tax form in exchange for a clothes ironing service. Another available service is to integrate ads and contracts to allow older people to rent rooms to students near universities, creating a symbiotic relationship. This measure intends to give students the possibility to have low cost or even free room in exchange for providing companionship to an elderly resident. With our present day economic European crisis leading to a dropout in higher education, this measure can reduce the financial burden of education while granting social support for older people. A further benefit fostered by this kind of relationship is to have seniors and young people share knowledge and experiences, learning from each other.

As the name implies, the call center category aims to provide user support through voice and/or video calls, to clarify any doubts and to provide social support, in addition to arranging for on-line psychologists to provide specialized help. It will also contain the remote device repairmen ability to assist in solving any issue reported by end users that does not imply a displacement.

The leisure category provides access to streaming TV, music streaming, games and other leisure activities. Games include activities to improve and stimulate memory and others to infer and correct deterioration in posture; while other activities appear to enhance and promote physical exercise. The leisure category will also provide a service focused on the elderly to allow musical expressiveness through motion, using only the resources available on an ordinary home computer.

The cultural category includes virtual visits to monuments and e-learning classes. This category will also include an experiences record, recording memories and knowledge, including handcrafts, recipes, folk medicine, proverbs, traditional agriculture methods, etc. It is extremely important to guarantee that the tacit knowledge acquired by seniors throughout the course of their life experience can persist across future generations.

All services are delivered as Software as a Service (SaaS), where providers publish their services on the catalogue and end user clients can consume these services. This leads to extreme simplicity from the client side by removing any installation and configuration. All services are available by accessing the platform via a web browser. This easy-to-use feature is of extreme importance considering the targeted population. In summary, the service catalogue will be implemented in the middleware platform and provide a service ecosystem organized into several main categories, all primarily focused on the needs of the elderly. To allow the addition of services and categories, the middleware platform has its own standards and ontologies, effectively creating an integration layer.

#### 3.2.2. System Administration

**Figure 3:**
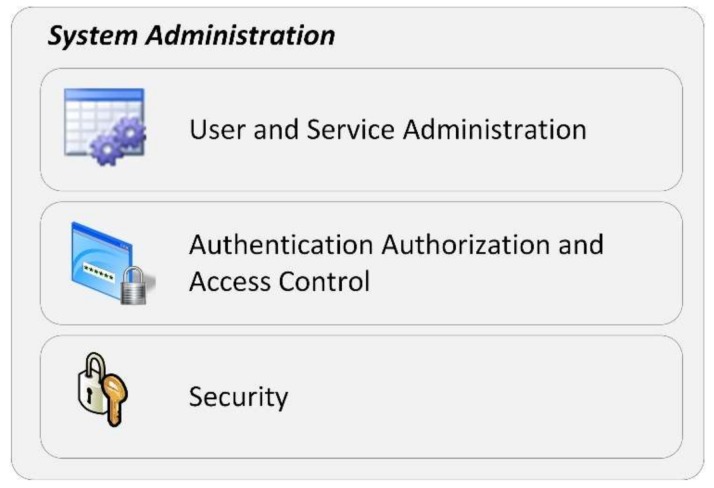
System administration.

System administration will be embedded in the middleware platform and will allow for the platform administration tasks to be conducted across a back office. These administration tasks are shown in Figure 3 and include issues such as user and service administration, security, authentication, authorization, access control, accounting, ciphering information, managing quasi-identifiers, data replication and balanced distribution methods through the cloud.

For instance, system administration holds metrics on the system users, what services they are using and whether they have adequate permission. After completing the registration process, the user’s login method is applied to gain access to a specific session.

Given the personal nature of the information, it is extremely important that only pseudonyms are sent through a secure middleware platform to service providers granting security. No sensitive data can be related to a service provider, therefore avoiding the possibility of linking sensitive information to a real person. This kind of association will only be given to authorized persons and requires user consent. For instance, the medical user may access the health records to analyze a certain situation. During the registration, users are also asked whether they want to allow certain information to be shared in eminent life or death situations and, if so, with whom the information should be shared. The persistence of sensitive data is also cyphered. Furthermore, due to the sensitivity of the information, the module needs to be able to create secure channels to connect end user clients and service providers, using the HTTPS (Hypertext Transfer Protocol Secure) protocol.

Additionally, and also related to security, session logs are stored for every action executed between connected users and available services.

Service registration will also be included in system administration, allowing for the addition of new services. This includes not only services developed by this research team, but also shelter services provided by other entities in a scalable mode.

#### 3.2.3. Event Management and Alert Deployment

Event management is integrated in the middleware platform, being responsible for event management and alert deployment. All the data collected by health monitoring parameters and other sensors present in the environment will be processed in the event management. Since some data require immediate processing and actions by sensors/actuators, while others have more relaxed constraints, the event management needs to provide several levels of priorities. For instance, events linked to imminent danger of death or serious injury need to be immediately processed. However, this quick reaction to an emergency needs to be balanced with power usage when battery-operated devices, namely sensors, are considered. Thus, battery-based sensors will periodically seek emergency situations, alternating between alert and sleeping states to preserve energy.

Other collected data are created from the interaction between the elderly and a multi-service platform (again, with user consent only) to establish behavioral patterns to detect deviations that may indicate some type of physical or psychological problem. For instance, if a user always accesses the platform and plays a specific game, a derivation of this behavior may indicate that something is wrong. In this context, a more careful study about behavioral deviations may suggest the existence of cognitive problems or physical limitations.

All generated alarms are then validated or classified as false positives, providing input to enhance the effective detection of alarm situations. The goal is to have the system learn the difference between false positives and real alarm situations for each user and therefore gain accuracy. Specifically, using the critical level of a generated situation as a weight, the sum of different inputs and associated weights generates a value that enhances an action. Each user is unique and may have different inputs and have different weights associated with each input. Therefore, in the initial phase the system needs to learn how to identify and further predict distress situations for the given user. The addition of several inputs, including biosensors and environment sensors, and the interaction with the platform is a significant gain in the ability to acquire accurate alarm situations and predict further distress. After acquiring initial data, several data mining techniques can be applied to obtain patterns. By combining information provided by biosensors and routines, including interaction with the platform and services, or where the elderly person is positioned, false positives can be almost eliminated. Reliability in dangerous situations or potential risk situations increases exponentially. In [49], we present a case study for detecting behavior patterns’ deviation in the elderly assisted living using eServices platform and the Cross-industry standard process for data mining (CRISP-DM) methodology. For this purpose, the biosensor, environment sensor and service usage are data sources to an ETL (Extract, Transform and Load) process, leading to a consolidated data warehouse where interesting patterns can be later recognized. Decision trees, built with the C5.0 algorithm, are used as a predictive data mining modeling method.

The communication architecture regarding events works as follows. Whenever a monitored reading is outside a configured threshold, a sensor node triggers a notification event to the eServices platform, through an appropriate message. The eServices platform reacts to the event message, triggering the appropriate response, which can range from simply logging the message to a full-scale response where emergency services are notified. Note that BAN-attached sensors communicate with the eServices platform through the communicator node, with environmental sensors that can directly reach the platform. Additionally, a sensor node receives its threshold values from the eServices platform, when it boots up. Threshold values can also be adjusted at runtime by the platform, to accommodate changes.

### 3.3. Services Provider

Service Provider will also be an input module for the middleware platform. Some of the services are going to be developed by this research team, while others may be provided by outside developers.

The middleware platform, where the elderly users can access any desired service, enables them to have only one access point to several services. This will simplify the end user’s interaction with the platform, as they will not need to memorize how to access each of the services that they need. Service providers will have to register their services in one of the existing categories or ask to generate a new one. Additionally, service providers will also need to use the middleware platform communication protocol. After the registration, icons and voice commands need to be created in order to grant accessibility from end users that subscribe to the service. Service providers also need to adhere to guidelines to maintain consistency across middleware platform services. This means that eServices will provide a digital repository with corporate identity design templates, also delivering an appropriate API.

## 4. Results and Discussion

In this section, we will provide details about the experiment that was carried out to validate our eServices platform, as well as discuss the obtained results from two complementary perspectives (i.e., quantitatively and qualitatively). Additionally, we also present a section specially focused on highlighting the important lessons learned after the implementation and validation of our eServices platform.

### 4.1. Experimental Setup

**Figure 4:**
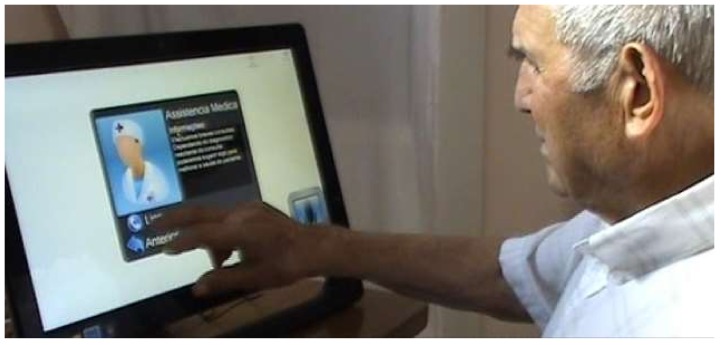
Service doctor appointment in the medical category.

**Figure 5:**
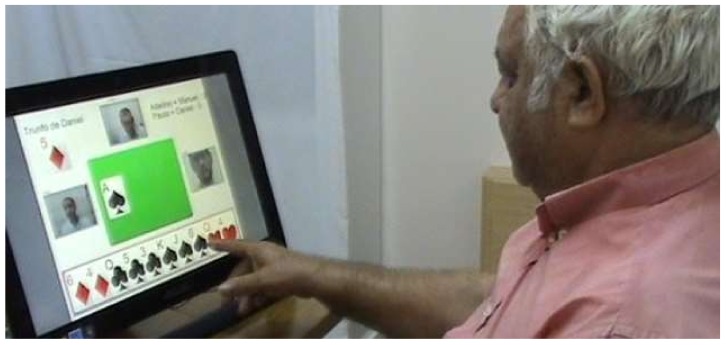
Service *sueca* card game in the leisure category.

To validate the proposal, a prototype was built, adding three services: one in the medical category, another in the leisure category and a third in the call center category. Figure 4 shows the service that was tailored to provide the ability for the elderly user to make a video call to a doctor (medical category). Figure 5 illustrates a four-player popular card game among the male senior population in Portugal, known as *sueca*, which was developed and added as a new service (leisure category). Finally, in the call center category, a service was added to allow sending an asynchronous voice message.

After a brief explanation of the system, acceptance tests were conducted with the consent of the users. First of all, the individuals were invited to perform a video call, play the *sueca* game and send a voice message. For each of these three tasks, we measured the time to perform each action and the errors/attempts made (following a quantitative perspective). The test protocol consisted of individually observing each subject, where only the user and the observer were present in an isolated room.

After the execution of this initial experiment, a semi-structured interview took place in order to obtain feedback about simplicity, further usage and importance in daily use (following a qualitative perspective). The demographic composition of the population sample was as follows.

Age population sample:

$$\begin{array}{c} \{33,\;35,\;37,\;52,\;64,\;65,\;65,\;68,\;69,\;71,\;71,\;73,\;73,\;75,\;76,\;76,\;78,\;80,\;80,\;81,\;81,\;83,\;84\}\\ \bar{x}=68\;years\;old \end{array}$$

Gender population sample:

$$\begin{array}{c} \{F,F,M,M,F,M,M,M,F,F,M,M,M,M,M,F,M,F,F,F,F,F,F\}\\ n=23\;\left(12\;females,\;11\;males\right) \end{array}$$

In the control questions of our interviews, we have asked about different health issues such as vision, hearing, motor skills and cognitive abilities. The most common problems manifested by participants were in relation to vision and hearing. Other less common issues were heart diseases, diabetes, arthritis, tremors, depression and cancer. Only one of the participants was identified as having early-onset Alzheimer’s.

Acceptance tests were conducted by asking users to accomplish the tasks present in Table 2.

### 4.2. Quantitative Results

**Figure 6:**
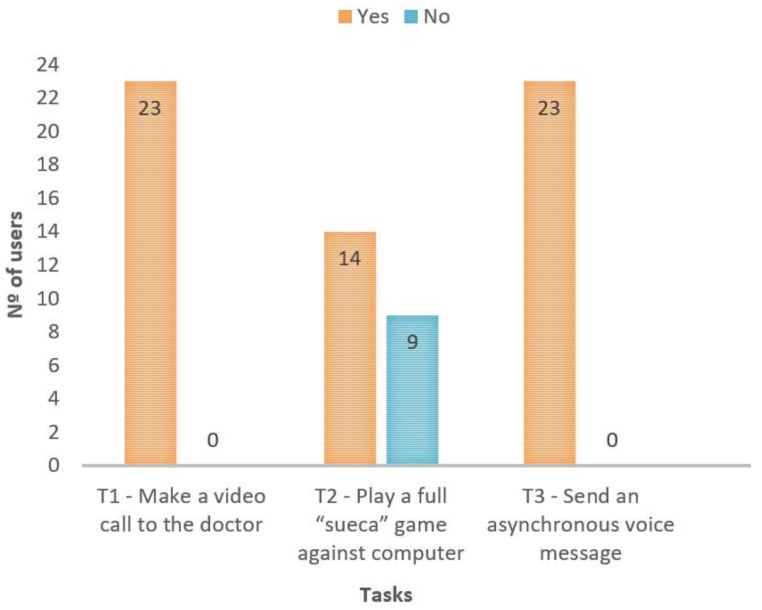
Results of acceptance tests.

The obtained results are presented in Figure 6, which indicates, for each task, whether the user could accomplish it. As Figure 6 shows, almost everyone was able to perform the tasks without any help or intervention. Nine elderly users were unable to accomplish the card game task because they were not familiar with the game itself. In other words, we verified during the acceptance tests that some users (despite not knowing how to play the *sueca* game) were able to interact with the platform (e.g., they succeed in sending a card without knowing which one), and the difficulty was related to the rules of the game, not to the interaction.

We selected some participants of approximately 30 years of age in order to compare the times required to accomplish a given task. We observed that the times to accomplish the proposed tasks between adults and seniors were very similar.

They experienced the touch screen and voice commands to perform the tasks.

### 4.3. Qualitative Discussion

Regarding the results obtained through the semi-structured interviews, all tests mentioned the system’s simplicity and their desire not only to repeat the experience, but to use the tested eServices on a daily routine. One of them saw an opportunity to make a free video phone call to his granddaughter in Brazil. Another one commented that it was like reliving old days, when he had more interaction with others. 

The main purpose of the interviews and the acceptance test was to prove the importance of the key features proposed in the previous section. The simplicity provided by the sole requirement of using a web browser and the possibility of accessing eServices through any device (e.g., personal computers or tablets) are clearly beneficial when dealing with seniors. Combining environmental sensor information with basic vital signs and the users’ interaction with the platform is another extremely important feature regarding previously-developed work. Adding this information for each individual allows a more assertive reactive and preventive response. The vital importance of the presented solution to improve the quality of life for seniors is evident from the results of the performed interviews.

### 4.4. AAL and Senior Population

After the careful study of the state of the art and the successful implementation and test of our eServices platform, it is appropriate to identify existing areas of concern and discuss some relevant aspects related to the appropriate development and delivery of useful systems dealing with AAL in the senior population.

- Lack of regard for the overall needs of the elderly: There has been a focus on physical motorization while neglecting social issues, such as social exclusion and isolation, which are extremely important concerns to the senior population;
- Complex and invasive: Solutions made for elderly citizens must be as simple as possible. The actions they need to perform must occur in a natural way and be integrated in their daily routine, intuitively, without the need for memorization. It is extremely important to consider the functional limitations of the elderly. Almost all of the solutions are too complex or have widespread services that are not combined as one. Any solution must be non-invasive, not interfering with the user’s daily routines;
- Lack of concern about the need for developing a solution that should be easily used by any person, regardless of prior Information Technology (IT) knowledge: The interactions between some solutions and end users do not assume that end users are technologically or functionally illiterate, which is still the case with some senior citizens;
- Absence of a user-centered design approach: The elderly are likely to offer some resistance regarding new technologies. To overcome this barrier, they must be involved in the process. They are the essential key for success. If they do not see themselves as a part of the solution and their expectations are not taken into consideration, it is predictable that they will not use it [50]. The elderly also have some difficulties in handling traditional inputs such as keyboards and a mouse. In addition, more recent devices are increasingly incorporating an interactive touch screen. Interaction between the user and the system must be simplified by eventually replacing traditional inputs by touch, such as voice recognition or other kinds of more intuitive interactions [51]. Furthermore, user needs and expectations are forgotten and often relegated to the background, disregarding the fact that the end users are the most important component, in terms of both what they expect and need. It is extremely important to carry out studies to obtain user feedback. Many solutions do not explore what seniors want and thus fail to provide what they need and expect;
- Unreliable and reactive: Regarding alerts, the high number of false positives in some solutions is still a problem. To ensure system reliability, it is mandatory to send alerts only in real situations. False positives can jeopardize the entire alert mechanism. To reinforce the identification of real alert situations, solutions must consider several inputs, not only by collecting bio and environmental data, but also by identifying user routines and deviations in behavioral patterns. This addition of data will also compel the system to be preventive and proactive, instead of being merely reactive. Indeed, it is vital not only to act immediately in imminent danger situations, but also to be able to identify potential hazardous risks and prevent their occurrence;
- Missing alert levels that provide an immediate response in imminent danger or mark other situations as low risk: This kind of classification must be done for each user individually. The system must learn and categorize patterns for specific users, allowing for several forms of response according to each level. Many solutions are standard, applying the same response for every individual;
- Security and ethical issues are often disregarded: Solutions must avoid quasi-identifiers and protect user identities from service providers. In addition, all sensitive information must be cyphered. Nevertheless, users must have access to their health data and be able to share it with their doctor or other care providers. Health data implicitly involves a bio profile (clinical history/medical records), such as medical appointments, prescriptions and medical exams taken by the subject. The user must be fully aware of and consent to sharing his/her bio profile with emergency staff in life/death cases if unconscious or in full dementia;
- Lack of standards and protocols lead to integration problems. Many solutions feel like separate pieces of the same puzzle. This significantly complicates the ability to add services and encapsulate all the services available for seniors in a middleware platform. To work around the lack of standards and protocols, solutions should provide integration to upload health data manually or automatically;
- Unscalable: In a dynamic scenario, scalability is also a feature to take into account. The system must be able to add new users, new services, new sensors and new service providers. All the architecture must allow for the addition of components in a transparent way;
- Not considering that the solution should be able to be used anywhere, whether the person is at home, at hospital or outdoors, in a rural or urban area: There must be mechanisms to ensure the service has high availability in order to have an always-on solution. It is extremely important to be able to provide any service, accessible from any location, by any device, at any time, to everybody: AnyN (Any service, anytime, anywhere, any device, any access, any people, etc.);
- Costs supported by the senior population: Many seniors cannot afford complex and complete solutions as a whole. It may seem a better proposal to have a modular solution with basic and additional modules to append as needed and financially feasible.

## 5. Conclusions and Future Work

This paper presents eServices, a solution to improve the welfare and quality of life of seniors. An important research work was performed by reviewing and verifying solutions that have aimed to attain the same goal. Some key features were identified as vital to be present in a solution to promote health and physical, emotional and psychic well-being and included in eServices.

eServices provides a novel service ecosystem mainly developed for the senior population. Its principal characteristics are scalability due to easy add-in services, simplicity achieved by a unique central access point to end users and by allowing interaction between end users and services to be intuitive, accessible and easy to use.

The main contribution of eServices is to allow the addition of several services directed to the needs of the senior population. These services deal with physical aspects by monitoring their basic vital signs, their normal environment and their daily routine patterns. This monitoring reassures family members and caregivers, who are notified according to predefined policy alerts. The elderly also develop feelings of security due to this assistance.

There are also services oriented towards social issues, aiming not only to avoid loneliness common among seniors, but also to promote their sense of usefulness by allowing them to contribute to society. Examples include services where they can record and share their life experiences and knowledge, or the neighbor-to-neighbor time bank approach, where seniors may do some volunteer work or trade services among themselves or with other society members.

Relating alert management, eServices stands out among other solutions by combining several inputs, such as information from BAN, environment sensors and interaction with the platform. By establishing routines and detecting pattern deviations, abnormal situations are more accurately detected and the possibility to solve a potential issue increases. Therefore, eServices distinguishes itself from other solutions by allowing a reactive and preventive response tailored to each specific user, with various levels of alert.

In line with our experiments and results, we built a functional prototype by adding three different but complementary services: one in the medical category, another in the leisure category and a third in the call center category. The implemented prototype was validated by semi-structured interviews and acceptance tests carried out with 19 seniors and four younger adults. Summarizing the obtained results, there are two aspects that were very appreciated by seniors: (i) the possibility of making free video phone calls and (ii) the fact that they felt they were reliving days from their past. They also demonstrated some surprise with themselves by seeing that they were easily able to fulfill the assigned tasks. Therefore, during the sessions, we observed a demystification of ICT usage. Sentences such as “who knew I was able to do this at my age” were uttered by several individuals. It was also relevant to observe that some of them enjoyed testing the solution so much that they preferred to continue experimenting instead of carrying on with other pleasurable activities like praying the rosary. All of the feedback allowed us to conclude that the seniors were able to easily use the implemented solution and expressed enthusiasm and interest in using eServices in their day-to-day activities. 

The solution was placed in the private home of eight elderly participants in a “production mode”. Concerning future work, the intention is to assemble information and apply data mining mechanisms to detect alarm situations and behavior pattern disorders. Some work has already been done with the CRISP-DM methodology to the eServices platform in order to predict abnormal behavior [49]. Moreover, new end user contributions will be collected to improve the means of interaction between the elderly users and the platform, enriching design guidelines directed toward the senior population.

## Figures and Tables

**Table 1:** Key features present in several solutions. PERSONA, Perceptive Spaces promoting Independent Aging; OASIS, Open architecture for Accessible Services Integration and Standardization.

	PERSONA	OASIS	AAL4ALL	eCAALYX	UniversAAL	eServices
Physical and social axes concern	√	√	√		√	√
Simplicity						√
Touch interaction	√	√			√	√
Voice recognition	√	√			√	√
Combination of text, voice and in icons					√	√
Adaptive and personalized interface					√	√
User-centered design approach		√	√		√	√
Reliability	√		√	√		√
Reactive response	√	√	√	√	√	√
Preventive response		√			√	√
Behavior patterns in alert equation					√	√
Security	√	√	√	√	√	√
Non-invasive						√
Scalability	√	√	√	√	√	√
Any service	√	√	√	√	√	√
Anytime	√	√	√	√	√	√
Anywhere	√	√	√	√	√	√
Any device	√	√	√	√	√	√
Any access	√	√	√	√	√	√
Any people	√	√	√		√	√

**Table 2:** Tasks belonging to the acceptance tests carried out.

Task Identifier	Task
T1	Make a video call to the doctor
T2	Play a full *sueca* game against computer
T3	Send an asynchronous voice message
